# Celiac Disease in Patients with Cystic Fibrosis-Related Bone Disease

**DOI:** 10.1155/2017/2652403

**Published:** 2017-11-02

**Authors:** Melissa S. Putman, Alexandra Haagensen, Isabel Neuringer, Leonard Sicilian

**Affiliations:** ^1^Division of Endocrinology, Boston Children's Hospital, Boston, MA, USA; ^2^Endocrine and Diabetes Units, Massachusetts General Hospital, Boston, MA, USA; ^3^Pulmonary Division, Department of Medicine, Massachusetts General Hospital, Boston, MA, USA

## Abstract

Both cystic fibrosis (CF) and celiac disease can cause low bone mineral density (BMD) and fractures. Celiac disease may occur at a higher frequency in patients with CF than the general population, and symptoms of these conditions may overlap. We report on two patients presenting with CF-related bone disease in the past year who were subsequently found to have concurrent celiac disease. Because adherence to a gluten-free diet may improve BMD in patients with celiac disease, this could have important implications for treatment. Clinicians should consider screening for celiac disease in patients with CF who have low BMD, worsening BMD in the absence of other risk factors, and/or difficult to treat vitamin D deficiency.

## 1. Introduction

Cystic fibrosis (CF) is one of the most common autosomal recessive disorders among Caucasian populations, affecting approximately one in 3000 live births. Life expectancy for patients with CF has increased dramatically over the past several decades, from approximately 29 years of age in the late 1980s to 41 years of age in 2015 [1]. As patients with CF live longer, endocrine complications such as CF-related bone disease are becoming increasingly prevalent. Children and adults with CF are at risk for low bone density and fractures due to multiple potential risk factors including vitamin D deficiency, delayed puberty, hypogonadism, pancreatic insufficiency causing malabsorption, compromised nutrition, glucocorticoid use, reduced physical activity, and possibly CF transmembrane conductance regulator (CFTR) dysfunction itself [2, 3]. Fractures, particularly of the ribs and vertebrae, can lead to significant morbidity in patients with CF.

Celiac disease is an autoimmune disease of the small intestine caused by sensitivity to dietary gluten resulting in intestinal malabsorption. Screening for celiac disease typically entails serologic testing of celiac-specific antibodies followed by pathologic confirmation with duodenal mucosal biopsy [4]. Celiac disease has been associated with compromised bone density in children and adults [5]. The symptoms of celiac disease may be difficult to distinguish from those related to CF, particularly in patients with malabsorption from pancreatic insufficiency. The combination of these two conditions may result in a significant insult to skeletal health.

We report two cases of young adults who presented to our CF Center Endocrine Clinic in the past year with CF-related bone disease and upon further evaluation were subsequently diagnosed with concurrent celiac disease.

## 2. Case  1

A 28-year-old white male with a history of CF (homozygous for the F508del mutation), CF-related diabetes (CFRD), and pancreatic insufficiency presented for evaluation of low bone density. Dual energy X-ray absorptiometry (DXA) scan showed low bone density with a significant decline in BMD compared to a previous scan obtained four years prior, as follows:

  Femoral neck: *Z*-score −2.6, decline in BMD by 13.3% over the preceding four years
  Total hip: *Z*-score −2.3, decline in BMD by 11.2%
  PA spine: *Z*-score −1.8, decline in BMD by 11.1%

The patient reported no significant oral or inhaled glucocorticoid use, delayed puberty, tobacco or alcohol use, or other risk factors for low bone density. He was compliant with pancreatic enzymes. He endorsed excellent dietary calcium intake, and his 25-hydroxyvitamin D (25[OH]D) level was at goal 57 ng/mL on cholecalciferol 3000 IU daily. His BMI at the time of evaluation was 22.1 kg/m2, which had improved after starting insulin for treatment of CFRD several months before. He reported a possible rib fracture occurring earlier in the year, though this was not confirmed on X-ray. Pertinent family history included an aunt with celiac disease.

Laboratory evaluation showed normal parathyroid hormone (PTH), calcium, phosphorus, alkaline phosphatase, renal function, and morning testosterone level. Screening tests for celiac disease showed a total immunoglobulin A (IgA) of 375 ng/dL (reference range 69–309 mg/dL) and an elevated anti-tissue transglutaminase (TTG) IgA level of 195.09 U/mL (reference range 0–15 U/mL). He was also found to have significant iron deficiency with a low ferritin level of 6 ug/L (reference range 20–300 ug/L) and a low transferrin saturation of 5%. He underwent endoscopy with duodenal biopsies, and pathology revealed villous blunting with intraepithelial lymphocytosis consistent with celiac disease. A gluten-free diet was recommended for treatment. Follow-up DXA scans after initiation of treatment are not yet available given that the diagnosis of celiac disease was made within the past year.

## 3. Case  2

A 28-year-old white male with a history of CF (homozygous for the F508del mutation), pancreatic insufficiency, and progressive pulmonary decline over the preceding 6 months presented for evaluation of bone health prior to lung transplantation. Pretransplant screening DXA scan showed low bone mineral density (BMD) with femoral neck *Z*-score −2.6, total hip *Z*-score −2.3, and PA spine *Z*-score −2.2. As with the first case, the patient reported no prior significant oral or inhaled glucocorticoid use, delayed puberty, or significant tobacco or alcohol use. He endorsed reduced physical activity over the year prior to his evaluation related to his advanced lung disease. He had never suffered any fractures. He reported robust dairy intake every day, but he had persistent vitamin D deficiency (25[OH]D level 26 ng/mL) despite being compliant with ergocalciferol 50,000 IU twice weekly. His BMI was low at 18.8 kg/m2, and he reported longstanding difficulty with weight gain despite taking pancreatic enzymes with all meals and snacks. He denied any family history of celiac disease or other autoimmune or gastrointestinal disorders.

Laboratory evaluation showed normal PTH, calcium, phosphorus, alkaline phosphatase, renal function, and morning testosterone level. Total IgA was 729 mg/dL and TTG IgA level was elevated at 82.60 U/mL. The patient was evaluated by gastroenterology but was deemed too ill to undergo confirmatory testing with endoscopy and duodenal biopsy. He was empirically started on a gluten-free diet. Shortly thereafter, he underwent lung transplantation and was treated with intravenous zoledronic acid to prevent bone loss and fractures in the posttransplant period.

## 4. Discussion

To our knowledge, these are the first reported cases of celiac disease diagnosed in patients presenting with CF-related bone disease. In both cases, celiac disease was discovered in young adults with CF who had significantly low BMD *Z*-scores by DXA, and one patient had a marked decline in BMD despite few other risk factors for progressive bone loss. CF and celiac disease are each associated with low bone density and increased fracture risk, and the combination of these two conditions may lead to even further compromise in bone health.

Ethnicity may predispose patients to both of these conditions, since celiac disease and CF tend to occur in Caucasian populations. Moreover, recent studies suggest that celiac disease may occur more frequently in CF patients than in the general population. In one study of 790 Scandinavian CF patients, 1.2% of CF patients were diagnosed with concurrent celiac disease, which was a prevalence rate of about three times higher than the general prevalence of celiac disease in this region [6]. Another study of Polish CF patients found an incidence of celiac disease of 2.13%, compared to 0.25% in the general population [7].

The signs and symptoms of celiac disease, such as poor weight gain and malabsorption, are nonspecific and may be difficult to distinguish from common symptoms of CF, particularly in patients with pancreatic insufficiency. In addition, vitamin D deficiency commonly occurs in both celiac disease and CF. In one of the reported cases, the patient had persistent vitamin D deficiency despite treatment with a very high dose of vitamin D (ergocalciferol 100,000 IU/week). Although not specific, persistent vitamin D deficiency in a CF patient on high dose supplementation may raise suspicion for concurrent celiac disease. In addition, one case was noted to have significant iron deficiency, which can be associated with both celiac disease and CF but potentially may be more severe in patients with both conditions.

In patients with celiac disease, adherence to a gluten-free diet may lead to improvements in bone density, whereas undiagnosed celiac disease can result in progressive bone loss and secondary osteoporosis [5]. For this reason, the diagnosis of celiac disease may have important implications on the management of patients with CF-related bone disease. Future longitudinal studies will be needed to determine if adherence to a gluten-free diet leads to significant improvement in BMD in patients with both celiac disease and CF.

In conclusion, patients with CF may be at higher risk for celiac disease than the general population, and combination of these two conditions may have a significant adverse effect on bone health. Clinicians should consider screening for celiac disease in patients with CF who have low BMD, worsening BMD in the absence of other risk factors, and/or difficult to treat vitamin D deficiency. Future studies will be needed to determine the effect of a gluten-free diet on bone health in patients with CF and celiac disease.

## Abbreviations

25(OH)D: 25-Hydroxyvitamin D
BMD: Bone mineral density
BMI: Body mass index
CF: Cystic fibrosis
DXA: Dual energy X-ray absorptiometry
IgA: Immunoglobulin A
PTH: Parathyroid hormone
TTG: Anti-tissue transglutaminase.
